# The genesis of adiabatic shear bands

**DOI:** 10.1038/srep37226

**Published:** 2016-11-16

**Authors:** P. Landau, S. Osovski, A. Venkert, V. Gärtnerová, D. Rittel

**Affiliations:** 1 Physics department, NRCN, Beer Sheva, 84190, Israel; 2 Faculty of Mechanical Engineering, Technion, Haifa 32000, Israel; 3 Chemistry department, NRCN, Beer Sheva, 84190, Israel; 4 Laboratory of nanostructures and nanomaterials, Institute of Physics, AS CR, v. v. i., Na Slovance 2, Prague, Czech Republic

## Abstract

Adiabatic shear banding (ASB) is a unique dynamic failure mechanism that results in an unpredicted catastrophic failure due to a concentrated shear deformation mode. It is universally considered as a material or structural instability and as such, ASB is hardly controllable or predictable to some extent. ASB is modeled on the premise of stability analyses. The leading paradigm is that a competition between strain (rate) hardening and thermal softening determines the onset of the failure. It was recently shown that microstructural softening transformations, such as dynamic recrystallization, are responsible for adiabatic shear failure. These are dictated by the stored energy of cold work, so that energy considerations can be used to macroscopically model the failure mechanism. The initial mechanisms that lead to final failure are still unknown, as well as the ASB formation mechanism(s). Most of all - is ASB an abrupt instability or rather a gradual transition as would be dictated by microstructural evolutions? This paper reports thorough microstructural characterizations that clearly show the gradual character of the phenomenon, best described as a nucleation and growth failure mechanism, and not as an abrupt instability as previously thought. These observations are coupled to a simple numerical model that illustrates them.

Adiabatic shear banding (ASB) is a synonym for a unique dynamic uncontrolled failure mechanism^1^,^2^. ASB implies a concentrated shear deformation mode that ultimately results in catastrophic failure after violent impacts or high-speed machining for example^2^.

The leading paradigm is that a competition between strain (rate) hardening and thermal softening determines the onset of the failure^3^,^4^,^5^. But more important is the fact that adiabatic shear is universally considered as an instability (material or structural), and therefore modeled on the premise of stability analyses^5^. In other words, ASB is hardly controllable or even predictable to some extent.

However, contrary to the current paradigm, it was recently shown that instead of thermal softening mechanisms, microstructural softening transformations such as dynamic recrystallization (DRX) are responsible for adiabatic shear failure^6^. Those transformations are dictated by the stored energy of cold work^7^, so that energy considerations can be used to macroscopically model the failure mechanism^8^.

Yet, one question persists, namely, what brings to the formation of a shear band? What are the initial mechanisms that will later lead to final failure? And most of all, is adiabatic shear failure an abrupt instability or rather a gradual transition as would be dictated by microstructural evolutions?

This paper reports fine scale microstructural characterizations that clearly show the gradual character of the phenomenon, best described as a nucleation and growth failure mechanism, as opposed to an abrupt instability, as thought until now. These observations are coupled to a simple numerical model that illustrates them.

Annealed, commercially pure α-titanium was used as a model system in this study. Previous work has shown that the fillet of dynamic shear specimens is a preferential locus for ASB formation and subsequent failure^6^. Therefore, the main idea was to dynamically deform a specimen by imparting a carefully controlled strain of 0.9*ε*_*f*_, *ε*_*f*_ being the strain to failure under given loading conditions. This value was chosen, knowing that DRX’ed grains were observed to form solely from this strain level and beyond in this material^6^. Scanning (SEM) and transmission (TEM) electron microscopy were used to characterize the specific microstructure in the fillet area.

## Results

**Figure 1:**
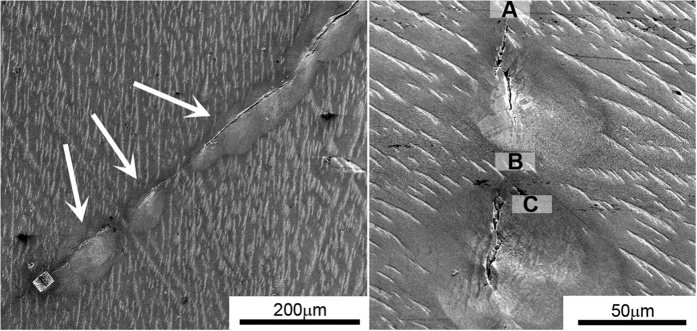
Scanning electron microscopy images of the cracks in the Compression Specimen (capitals) (SCS) gauge fillets. (**a**) Discontinuous crack segments (arrowed). (**b**) Two separate crack segments separated by ~40 μm. The transparent white boxes indicate the location from which samples were lifted out, perpendicular to the image plane.

Figure 1 shows SEM images of the investigated fillet area. While the specimen is not broken, Fig. 1a indicates the presence of cracks in the gauge fillet. Higher magnification (Fig. 1b) reveals that the crack consists of several discontinuous segments spaced 40–50 μm apart. Three types of samples were extracted for microstructural characterization, using focused ion beam (FIB) - see supplementary information: A. Right next (<5 μm) to the crack-tip, B. Uncracked ligament between crack tips, about 20 μm from the tip, and C. Close proximity to the crack (5 μm aside from the crack segment). All samples were taken perpendicular to the crack propagation direction. The microstructure of each specimen was characterized, as reported next.

### Right next to the crack-tip

**Figure 2:**
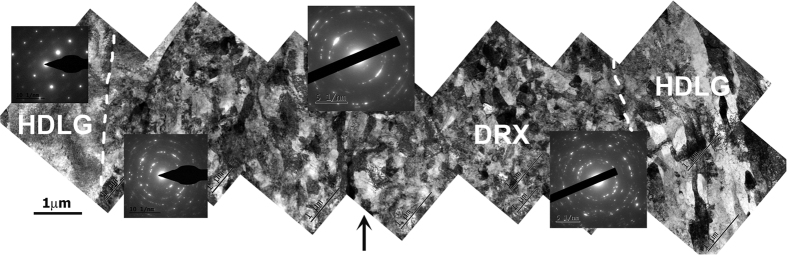
Dynamically recrystallized grains at the tip of a crack (box A in Fig.1b). The crack is perpendicular to the image. The crack tip location is indicated with the black arrow, at the mid-bottom of the image. The dense DRXed region extends roughly 5 μm to each side, with dashed lines delineating the extent of the DRXed region. Beyond that, heavily deformed large grains (HDLG), with typical features such as subgrains and twins, are observed. Corresponding selected area diffraction patterns show ring patterns in the DRXed regime, and single crystal patterns away.

Figure 2 shows the massive presence of dynamically recrystallized (DRX) nanograins (≤100 nm in diameter). The width of the DRXed region is ~10 μm across the crack, and the nano-grains occupy more than 75% of the entire area. Beyond that region, on both sides of the crack tip, the microstructure is typical of heavily deformed Ti, i.e. elongated dislocation cells and subgrains within large grains, but nanograins are no longer observed. Twins are also present in the heavily deformed regions. Interestingly, grains that deform by the formation of subgrains/dislocation cells do not show twins. This result is consistent with previous observations reported in ref. 6 (box A in Fig. 1b).

### Between crack tips

**Figure 3:**
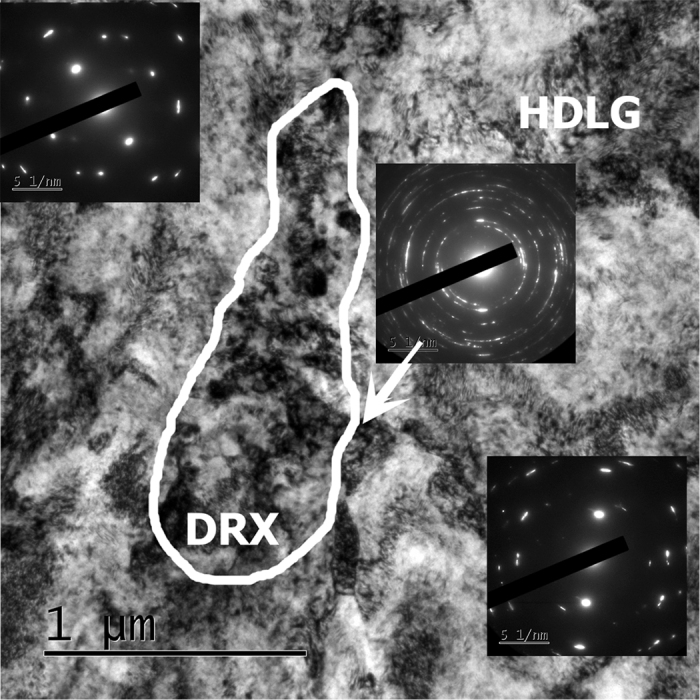
Sample between the cracks: heavily deformed Ti matrix and small DRX’ed regions and highly deformed large grains (HDLG). A single DRX island is bounded by a white line. Corresponding diffraction patterns are inserted in the image.

Here, the microstructure consists essentially of heavily deformed large grains (HDLG) with occasional twins and dislocation cells/subgrains (Fig. 3). Within this region, individual islands of DRX are discernable. The DRX’ed islands nucleate within the deformed matrix. In a single FIB’ed lamella (~10 × 5 μm^2^), no more than two DRX’ed islands were observed. Given that each island occupies ~0.5 μm^2^, this barely represents 2% (area density) of the whole area. The presence of sparse DRX’ed grains is supported by the ring diffraction patterns inserted in Fig. 3, that are typical of nano-crystalline materials, within the deformed matrix (box B in Fig. 1b).

### 5 μm aside from the cracks

**Figure 4:**
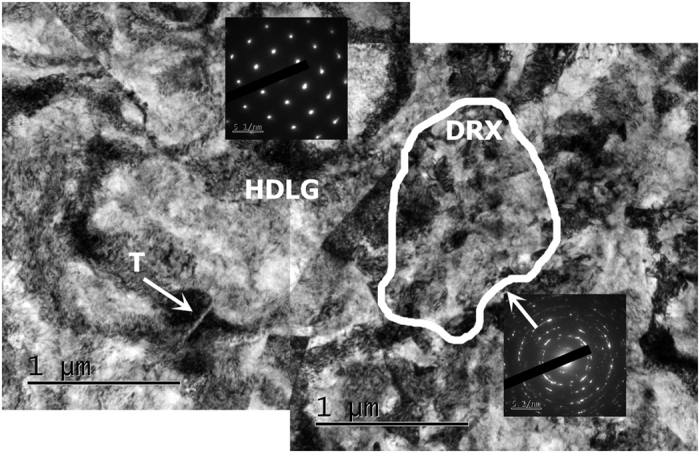
Sample C, ~5 μm aside from the crack. Heavily deformed microstructure with a DRX island within it (marked by the white line), similar to the microstructure observed between the cracks. A twin is arrowed in the heavily deformed region. Corresponding diffraction patterns are inserted in the image.

This region is characterized by heavily deformed large grains (HDLG), subgrains, twins and sparse DRX’ed (DRX) islands, as shown in Fig. 4 with corresponding diffraction patterns. This microstructure bears a definite resemblance to that observed between the cracks (compare Figs 3 and 4) (box C in Fig. 1b).

## Discussion

To summarize the main findings of this microstructural characterization, the immediate vicinity of the crack tip is characterized by densely-packed DRXed nano-grains, whose concentration drops dramatically in the un-cracked ligaments. Those observations reveal that failure (specimen fracture) occurs by the formation of *discontinuous cracks* along the fillet, spaced several tens of micrometers apart. In both the uncracked ligaments and roughly 5 μm away from the crack tips, the microstructure consists of heavily deformed large grains with typical features such as dislocations, dislocation cells, subgrains and twins. Within these regions DRX’ed islands are seldom observed, and they appear to nucleate as irregular low-density clusters. The area density of DRXed nanograins at the crack tip is almost 2 orders of magnitude larger than that that of the sparse DRXed islands, some 5 μm away from the crack-tip.

Those observations reveal that the microstructure evolves *gradually* from sparse DRX islands within a deformed matrix, to massive DRX areas in the immediate vicinity of the (future) crack-tip. Rittel *et al.*^9^ showed in earlier work that DRX precedes the formation of ASB instead of being its outcome as commonly believed. These authors proposed that the dynamically recrystallized grains cause local softening of the surrounding hardened matrix, thereby providing the necessary perturbation whose growth is the shear band^9^.

In this work, we show that regions with a local high density of DRX are associated with cracking, while other regions in which DRX density is small or not developed, simply don’t crack. This not only strengthens the earlier claim that DRX precedes shear localization, but also indicates that the phenomenon is likely of a percolative nature in terms of density (see molecular dynamics simulations of Chen *et al.*^10^).

Beyond the mere elucidation of the sequence of events leading to failure, the present results shed new light on the physical mechanisms leading to shear localization failure.

Instead of being an abrupt instability as long believed, the present results clearly show that the nucleation, multiplication and growth of DRX’ed islands are all prerequisites for local fracture. Those islands of a high DRX density exhibit no (or little) strain hardening, as opposed to the surrounding matrix, and thus localize the deformation within them. The arising incompatibility between the high density DRX region and its surroundings serves as a source for void (crack) nucleation. The mechanism presented here is of a cascading nature. When micro cracks are formed in high density DRX regions, the connecting ligament experiences intense plastic deformation, thus accelerating the DRX process, leading in turn to additional local damage. Final fracture is achieved due to the coalescence of the observed micro cracks (damage).

In other words, the genesis of an adiabatic shear band is *simply another form of ductile failure*, controlled by the nucleation, growth and coalescence of voids and micro cracks. Here, the initiation of the failure process is driven by DRX’ed nanograins. The evolution of the DRX’ed nanograins is initially controlled by the heterogeneity in energy storage capability of the material (see Benzerga *et al.*^11^), and at later stages is driven by the cascading process of damage evolution. As such, it no longer seems to have the features of an abrupt and uncontrolled transition which characterizes an instability. It should be noted that earlier work has shown discontinuous cracked segments in adiabatic shear bands, as for example in Teng *et al.*^12^, but those were not subjected to the thorough microstructural characterization at a very fine scale as reported here, revealing the nature and sequence of mechanisms leading to ultimate fracture.

The experimental observations for the genesis of failure by adiabatic shear banding are further illustrated by a simple micro-mechanical model based on ref. 6. The full model is detailed in the supplementary information section, while its main features are summarized here. The model considers only DRX (excluding twinning as in ref. 6) since twinning postpones DRX. The nucleation and growth of DRX islands governed by the stored energy of cold work, and its kinetics are described using the Johnson-Mehl-Avrami-Kolmogorov equation^13^.

The yield surface of the material is taken as a composite rule of the two phases considered here, i.e. the coarse-grained matrix material and the DRX’ed nanograins. The strain hardening behavior of the matrix materials obeys a power law (i.e. 
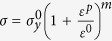
). The DRX’ed nanograins were assumed to be non-hardening as they poses a very low density of dislocations. Moreover, as suggested by Bouaziz *et al.*^14^, there exists a critical grain size below which dislocation storage drops dramatically. The flow stress of the DRX’ed nanograins embedded in the coarse grained material was assumed to be roughly 30% higher than the yield stress of the coarse grained titanium, following the well know Hall-Petch rule. To capture the main features of the failure mechanism, a damage evolution function was introduced, following the formalism of Nahshon and Hutchinson^15^.


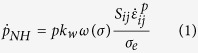


Here 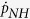
is the rate of damage growth and 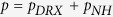 is the overall damage. *S*_*ij*_ is the deviatoric part of the stress tensor, 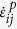 represent the plastic strain rate tensor component and *σ*_*e*_ is the effective (Mises) stress. The function *ω(σ*) represents the stress state in the system and is a function of the third invariant *J*_3_ and the Mises stress as shown in Equation (2). *k*_*w*_ is a fitting parameter.


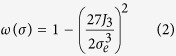


Equation (1) is structured such that it only allows for growth of damage from a pre-existing nucleus, and only when the stress state is predominated by shear, and decays to zero for a symmetric stress state. For a more realistic situation, damage nucleation (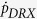) is assumed to originate from areas in which a threshold of DRX volume fraction 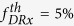 is observed, as described by Equation (3), similar to the stress and strain nucleation criterions often used in modeling ductile fracture^16^.


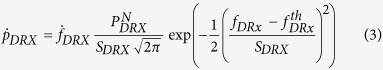


Here, 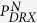 is the maximum value of damage that can nucleate by the formation of new DRX nanograins and 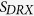 is controlling the rate of nucleation. Finally, the degradation of flow stress with evolving damage (Φ) is given by Equation (4).


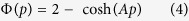


with A being a fitting parameter.

**Figure 5:**
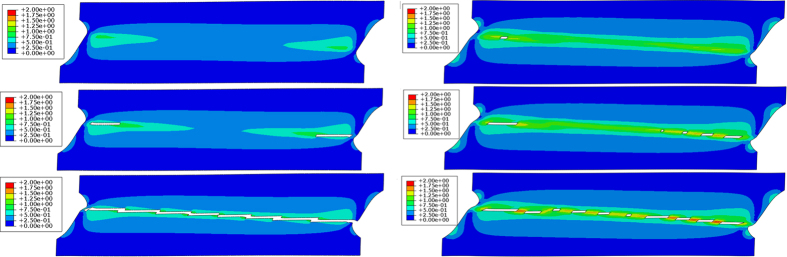
Contour plots of plastic strain for two cases. Case A, where the stored energy required for DRX nucleation (U_DRX_) is *homogenous* (**a**–**c**). Case B (**d**–**f**), where U_DRX_ is *heterogeneous* and correspond to a Gaussian distribution around the value used in case A. The plastic strain distribution and damage evolution (white regions) are presented for three snapshots, showing the continuous evolution of damaged sites in case A, compared with the spurious damage apparent for case B.

Finite elements calculations were carried using the commercial finite element package ABAQUS/Explicit^17^. The coupled plasticity and damage model was implemented in a user defined material subroutine (VUMAT) using the rate tangent stress update algorithm^18^. Boundary conditions were chosen such as a state of simple shear prevails throughout a predefined gage section. To demonstrate the catastrophic effect of DRX evolution on the overall mechanical response, two cases were considered. In case A, all elements are assumed to nucleate DRX upon reaching *the same* critical value of stored energy (U_DRX_). In case B, the heterogeneous nature of the material is captured by choosing the value of U_DRX_ from the left half of a Gaussian distribution centered around the value chosen for the uniform case. The damage evolution for the two cases is presented in Fig. 5.

The results of our numerical exercise clearly show the progressive discontinuous character of fracture within the localization band for the more realistic case B, as opposed to spatially continuous damage for case A. Furthermore, it appears from case B that fracture can take place in spurious locations along the potential localization band, as dictated by the materials propensity to nucleate DRX nanograins, just like observed experimentally.

The present experimental and numerical results suggest that for successful prediction and control of the adiabatic shear process, microstructural concepts must be taken into account. Furthermore, the framework of ductile fracture, which has shown great success in correlating microstructures with failure mechanisms should be used to further study the role of different material heterogeneities on the formation of adiabatic shear localization. We propose that a combined mechanical and microstructural effort should be taken to design microstructures and textures in such a way that a materials’ tendency to fail by adiabatic shear failure could be controlled.

To conclude, this work has uncovered the very early stages of the shear localization, which consist of the nucleation, multiplication and growth of discontinuous islands of dynamic recrystallization. At a certain stage, those islands develop microcracks which are bridged by additional islands of DRX, whose formation is accelerated by the local intense strains. This discontinuous evolving damage mechanism cannot be considered as an abrupt instability as previously thought, and belongs instead to the damage nucleation and growth type of fracture.

As a general final remark, it can be noted that now that the microstructurally related damage mechanisms are well defined, one can think of new strategies for impact toughening of new materials.

## Methods

Dynamic shear experiments were performed on Ti shear-compression specimens (SCS)^19^,^20^ with 10 mm diameter, 2.5 mm gauge thickness, and 2 mm gauge width. This unique geometry enforces a state of dominant shear in the gauge section of the specimen^19^,^20^, while a mild stress concentration exists in the fillet of the groove thus dictating the locus for (forced) shear band formation. The dynamic experiments were performed using a split Hopkinson pressure bar (Kolsky) apparatus^21^ at a strain rate of 7000 sec^−1^. The specimens were deformed to 0.9*ε*_*f*_, *ε*_*f*_ being the strain to failure, using stop rings. Specimen preparation was done by standard lift-out procedure in a Dual-beam Focused Ion Beam (FIB) which allowed for the careful extraction of samples from specific regions of interest in the fillet area.

Thorough microstructural characterization was performed using diffraction contrast in a Tecnai TF20^TM^ transmission electron microscope.

## Additional Information

**How to cite this article**: Landau, P. *et al.* The genesis of adiabatic shear bands. *Sci. Rep.* **6**, 37226; doi: 10.1038/srep37226 (2016).

**Publisher’s note:** Springer Nature remains neutral with regard to jurisdictional claims in published maps and institutional affiliations.

## Supplementary Material

Supplementary Information
