# Supplementary material for: The genesis of adiabatic shear bands
Source: Sci Rep. 2016 Nov 16;6:37226. doi: 10.1038/srep37226 (PMC5110954; doi:10.1038/srep37226)
Supplement: Supplementary Information [file srep37226-s1.pdf]

Supplementary information

The genesis of adiabatic shear bands

P. Landau, S. Osovski, A. Venkert, V. Gärtnerová, D. Rittel

Sample extraction

Samples were extracted from the ASB (adiabatic shear band) to perform a careful examination of the local spatial microstructural evolution. The extraction of TEM (transmission electron microscope) samples by focused ion beam (FIB) allows a precise selection of the electron-transparent area, while a typical lamella size is 7x10μm^2, with a typical thickness of 100nm, as illustrated in Figure S1a.

Figure S1b shows a low magnification ion beam image of the surface of the sample. 3 different crack segments are observed, 40µm apart. The black rectangles indicate the locations from which 3 FIB samples were extracted. Figure S1c is a schematic 3D view of Figure S2b. Sample A was extracted from the crack tip, where the tip indicates the middle of the FIB'ed lamella. Sample B was extracted from the region between the crack segments, perpendicular to the crack propagation direction and symmetric with respect to the axis of the ASB, as indicated by the dashed line between the cracks in Figure S1c. Sample C was extracted >5µm aside from the crack.

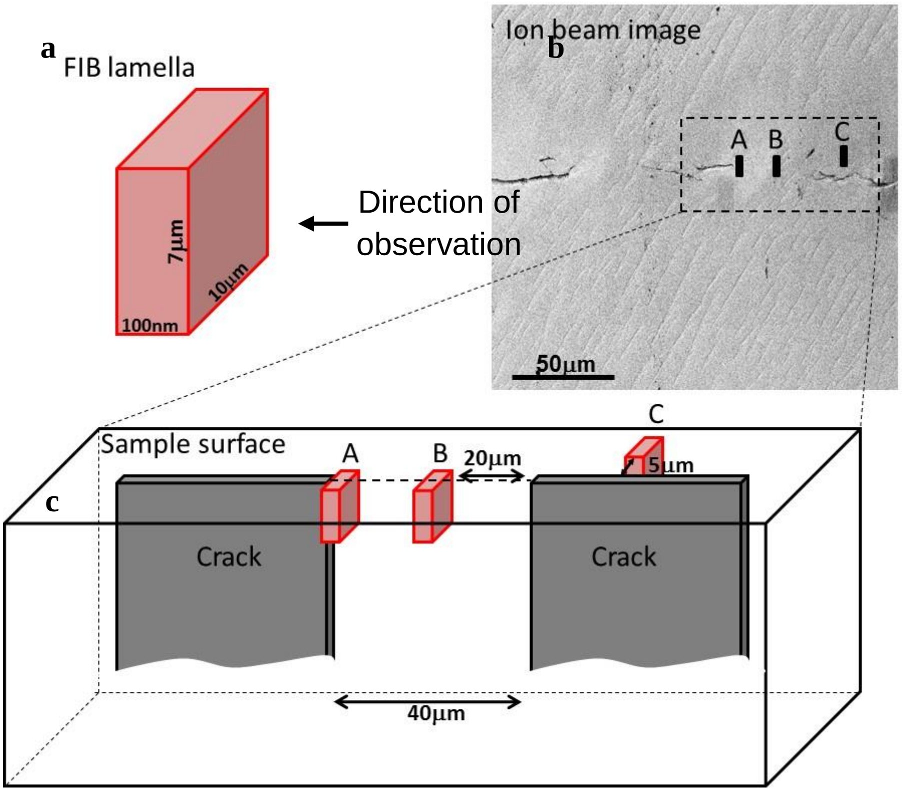

Figure 1S: a. FIBed lamella size and view of observation. b. Ion beam image showing the location from which FIBed samples were milled. c. 3D schematic illustration of the sample and the location from which samples were extracted.

The Micromechanical model

The mechanical model used is based on the experimental findings in [6]. Two hardening mechanism were considered:

a. A power law hardening law was used to describe the fraction of the material which has not recrystallized.
b. The portion of the material which has recrystallized is assumed to be non strain hardening.

The viscoplastic response of the material was modeled using:

$$\dot{\varepsilon}^{p} = \dot{\varepsilon}^{0}\left[\frac{\sigma_{flow}}{g\left(\varepsilon^{p}\right)}\right]^{1/n} \qquad (S1a)$$

$$g_0\left(\varepsilon^{p}\right) = \left(1 - f_{drx}\right)\sigma_y^0\left(1 + \frac{\varepsilon^{p}}{\varepsilon_0}\right)^{m} + f_{drx}\sigma^{drx} \qquad (S1b)$$

$$g\left(\varepsilon^{p}, T\right) = g_0\left(\varepsilon^{p}\right)\left(1 - \left(\frac{T - T_0}{T_m - T_0}\right)^{r}\right)\Phi \qquad (S1c)$$

with $g\left(\varepsilon^{p}\right)$ being a rate-independent yield surface. The first term in $g\left(\varepsilon^{p}\right)$ refers to the flow stress of the coarse-grained Titanium, while σ^{drx} in the second term is to be understood as the flow stress at which DRX deforms. ε_0 is taken to be the total strain at first yield. The contribution of each mechanism to the overall behavior is taken to follow a composite rule. Thermal softening is assumed to follow a power law behavior where T_0, T_m are the initial and melting temperature respectively. Φ is the softening induced by damage evolution (Equation 4). Finally, the rate dependence of the flow stress is given by the Equation (1a). Here, $\dot{\varepsilon}^{p}$ is the plastic strain rate, ε^{p} being the plastic strain and n is a rate-sensitivity constant, while $\dot{\varepsilon}^{0}$ is a reference strain rate.

The evolution equation describing the DRX formation is given by

$$f_{drx} = \begin{cases} 0 & ; \; U < U_{drx} \\ 1 - \exp\left(-k_{drx}\left(\frac{U - U_{drx}}{U_{drx}}\right)^{n_{drx}}\right) & U \geq U_{drx} \end{cases} \qquad (S2)$$

Where k_{drx}, n_{drx} are fitting parameters and U, U_{drx} are the stored energy and threshold energy for DRX formation respectively. The evolution of stored energy is given by

$$U\left(\varepsilon_p\right) = \left(1 - f_{drx}\right)\left(1 - \beta\right)\int_0^{\varepsilon_p}\sigma_{flow}\,d\varepsilon_p \qquad (S3)$$

β is the assumed Taylor Quinney coefficient of the coarse grained material.

Temperature in Equation (2) was calculated using the first law of thermodynamics:

$$\left.\begin{aligned} W - U &= \int \sigma_{flow} d\varepsilon_p - U = Q \\ \Delta T &= \frac{Q}{\rho C_p} \end{aligned}\right\} \qquad (S4)$$

With ρ being the material density and C_p the material's heat capacity.

The stress update was done using a forward gradient as a first approximation followed by Newton-Raphson iterative scheme to update all state variables. The geometry presented in Figure S2 was used to induce a state of simple shear. Calculations were carried out for a 2D model using 4-node bilinear elements with reduced integration. The nominal element size used was $35\mu m$. The model parameters are summarized in Table S1.

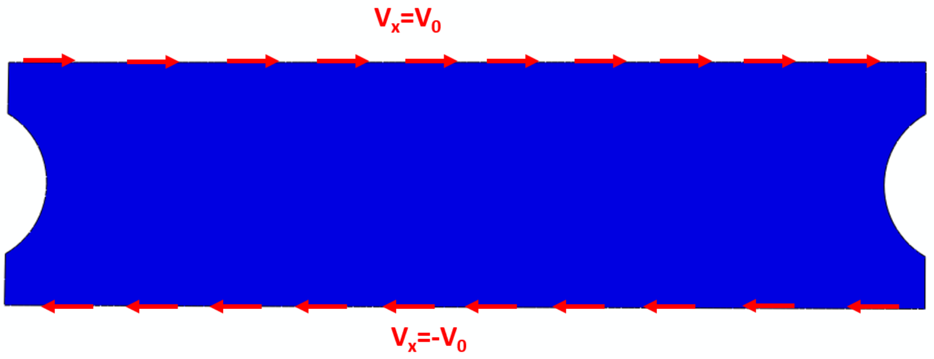

Figure S2: The geometry and boundary conditions used for the simulations presented in Figure 5. V_0 correspond to 3m/s and the overall gauge length was chosen to be 5mm.

Table S1. Model parameters

**Property**	**Value**	**Source**
E	116GPa	Young's modulus
ν	0.3	Poisson's ratio
U^*	$72\left[MPa/m^3\right]$	Threshold energy for the onset of the recrystallization
σ_y^0	$500[MPa]$	Yield stress
σ^{drx}	$630[MPa]$	Flow stress of the non-hardening recrystallized phase
m	0.075	Hardening power law coefficient
β	0.6	Taylor Quinney coefficient
C_p	$540[J/KgK]$	Heat capacity
k_{drx}	0.5	JMAK parameter
n_{drx}	2	JMAK parameter
f_{drx}^{th}	0.05	DRX volume fraction threshold for damage nucleation
k_w	2	Nahshon-Hutchinson damage parameter
P_{drx}^N	0.04	Maximum value of damage from nucleation events
S_{drx}	0.15	Damage nucleation parameter
A	0.08	Damage function parameter
$\dot{\varepsilon}^0$	$1000[1/s]$	Reference strain rate
n	0.053	Strain rate sensitivity
